# Supplementary material for: Contribution of Serum Cytomegalovirus PCR to Diagnosis of Early CMV Primary Infection in Pregnant Women
Source: Viruses. 2022 Sep 28;14(10):2137. doi: 10.3390/v14102137 (PMC9608498; doi:10.3390/v14102137)
Supplement: Supplementary file 1 [file viruses-14-02137-s001.zip › viruses-1929566-supplementary.pdf]

Figure S1: Flowchart of population study

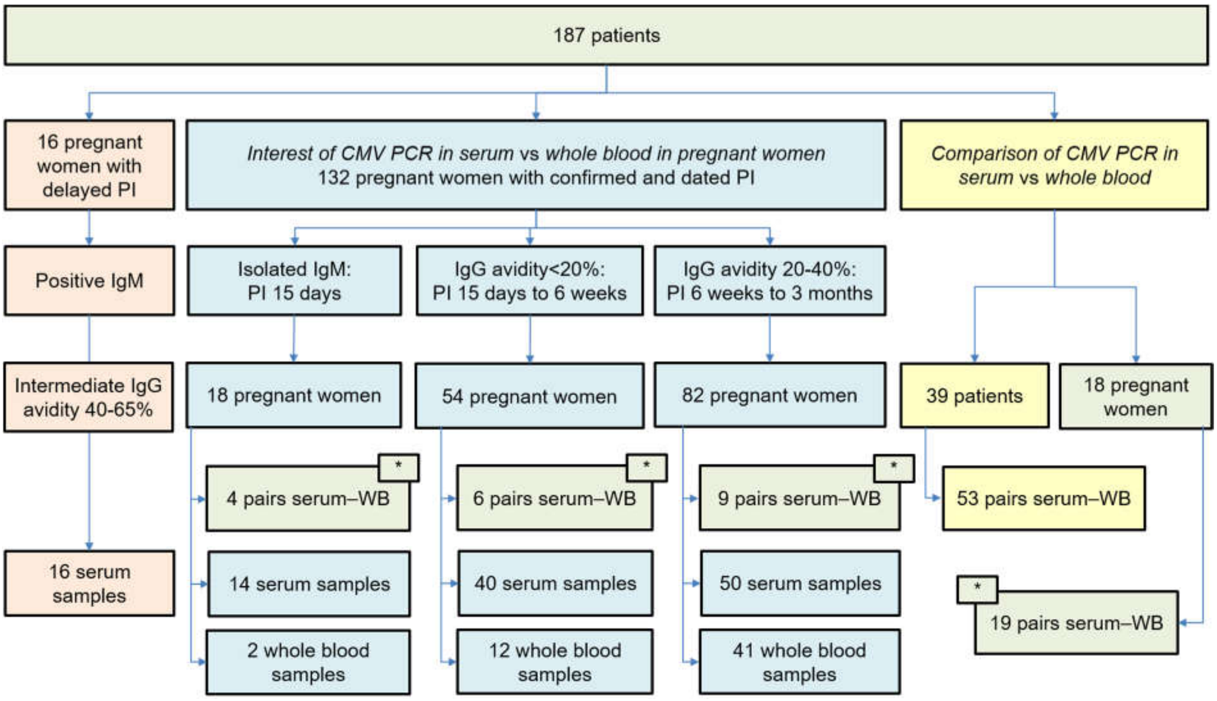

Our population study of 187 patients is splited in three categories:

- 16 pregnant women (orange) with delayed PI (more than one year): 16 serum samples with positive IgM and intermediate IgG avidity index (40-65%)

- 132 pregnant women (blue) to determine sensitivity of CMV PCR both in serum and in whole blood according to delay after CMV primary infection:
    - 123 serum samples
    - 74 whole blood samples

- 39 patients (yellow) + 18 pregnant women (green) with 72 paired serum and whole blood samples to compare quantitative CMV PCR in serum and whole blood

Boxes in green represent pairs of serum-whole blood from 18 pregnant women, both included in analysis of the two categories.

WB: whole blood

Figure S2: Correlation of quantitative CMV PCR between serum and whole blood

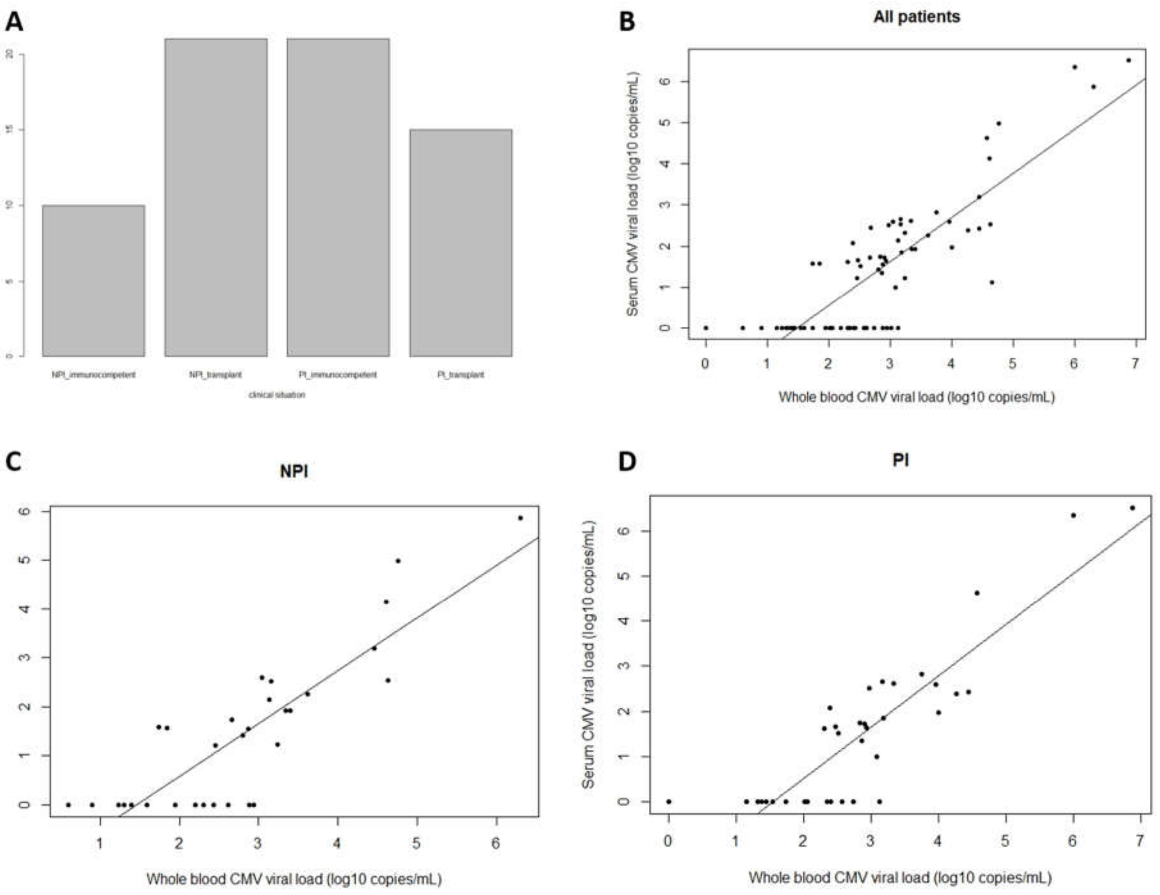

A: Repartition of the 72 pairs of serum-whole blood according to their clinical situation:

- 10 pairs from 10 immucompetent patients with CMV NPI
- 21 pairs from 15 transplanted patients with CMV NPI
- 22 pairs from 20 immunocompetent patients with CMV PI
- 15 pairs from 9 transplanted patients with CMV PI
- 4 pairs from 3 patients without classification

B: Plot of correlation between CMV viral load in serum *vs* whole blood for all patients, with linear regression line. PCR undetectable in serum were plotted at 0. R^2 was estimated at 0.71.

C: Plot of correlation between CMV viral load in serum *vs* whole blood for 25 patients with CMV NPI, with linear regression line. PCR undetectable in serum were plotted at 0. R^2 was estimated at 0.73.

D: Plot of correlation between CMV viral load in serum *vs* whole blood for 29 patients with CMV PI, with linear regression line. PCR undetectable in serum were plotted at 0. R^2 was estimated at 0.77.

NPI: non primary infection
PI: primary infection
